# Three-dimensional structure of the basal lamella of the middle turbinate

**DOI:** 10.1038/s41598-021-97331-2

**Published:** 2021-09-09

**Authors:** Márton Eördögh, Gábor Baksa, András Grimm, László Bárány, Örs Petneházy, Robert Reisch, Henry W. S. Schroeder, Hans Rudolf Briner, Werner Hosemann

**Affiliations:** 1 Department of Neurosurgery, University Medicine Greifswald, Sauerbruchstraße, 17475 Greifswald, Germany; 2 Department of Anatomy, Histology and Embryology, Semmelweis University, Budapest, Hungary; 3 Department of Neurosurgery, University Hospital Erlangen, Erlangen, Germany; 4 Justanatomy Ltd, Kaposvár, Hungary; 5 Endomin – Centre for Endoscopic and Minimally Invasive Neurosurgery, Zurich, Switzerland; 6 Center for Otorhinolaryngology, Head and Neck Surgery, Zurich, Switzerland; 7 Department of Otorhinolaryngology, Head and Neck Surgery, University Medicine Greifswald, Greifswald, Germany

**Keywords:** 3-D reconstruction, Anatomy

## Abstract

The middle turbinate’s basal lamella (3BL) is a variable landmark which needs to be understood in endoscopic transnasal skull base surgery. It comprises an anterior frontal and a posterior horizontal part and appears in its simplest depiction to be “L”-shaped, when viewed laterally. In this study we analyzed its 3D morphology and variations focusing on a precise and systematic description of the anatomy. CBCTs of 25 adults, 19 cadavers and 6 skulls (total: 100 sides) were investigated with the 3DSlicer software, creating 3D models of the 3BL. We introduced a novel geometrical classification of the 3BL’s shape, based on segments. We analyzed their parameters and relationship to neighboring structures. When viewed laterally, there was no consistent “L”-shaped appearance of the 3BL, as it is frequently quoted. A classification of 9 segment types was used to describe the 3BL. The 3BLs had in average of 2.95 ± 0.70 segments (median: 3), the most frequent was the horizontal plate (23.05% of all segments), next a concave/convex plate (22.71%), then a sigma plate (22.37%). Further types were rare. We identified a horizontal plate in 68% of all lateral views whilst 32% of the 3BLs were vertical. A sigma–concave/convex–horizontal trisegmental 3BL was the most common phenotype (27%). Globally, the sigma–concave/convex pattern was present in 42%. The 3BL adhered the ethmoidal bulla in 87%. The segmenting method is eligible to describe the 3BL’s sophisticated morphology.

## Introduction

Transnasal endoscopic surgery has become the basis of the management of paranasal sinus pathologies and skull base lesions. The expansion of indications for surgery led to the modification of surgical corridors, where the ethmoid bone is increasingly in focus^1–4^. Any surgical corridor addressing the ethmoidal cells must consider aspects of nasal physiology on the basis of intimate knowledge of ethmoidal microanatomy. In this aspect, one of the most important landmarks is the three-dimensional (3D) structure of the middle turbinate with its 3rd basal lamella (3BL)^5,6^. It is one of the lamellas which arise from folds (ethmoturbinates) on the lateral wall of the nasal cavity during intrauterine development (Table 1)^5^.

**Table 1:** Ethmoturbinate development.

Ethmoturbinate	Derivative of ascending branch	Derivative of descending branch
1	Agger nasi	Uncinate process
2	Ethmoidal bulla	
3	3rd basal lamella (of the middle turbinate)	
4	4th basal lamella (of the superior turbinate)	
5	5th basal lamella (of the supreme turbinate, if exists)	

Each ethmoturbinate is divided to an anterior ascending (ramus ascendens) and a posterior descending branch (ramus descendens).

Four main features of the 3BL are commonly accepted. Firstly, the MT’s skull base attachment line is composed of segments in the sagittal, frontal and horizontal axis; the 3BL is related to the frontal and horizontal ones^7–10^. Secondly, in a lateral view, the 3BL is described crudely as an “L”-shaped bone plate with an anterior frontal (vertical) and a posterior horizontal part^7,10^. Thirdly, the individual expansion of neighboring anterior and posterior ethmoidal cells (“struggle of the ethmoid”) is associated with alterations of the 3BL’s shape^7,10^. Lastly, each 3BL is unique^11^. Consequently, the identification of the 3BL and its optimal entry zone during ethmoidectomy can be challenging.

However, there are hardly any studies which go beyond these statements. Most reviews only examine the 3BL on triplanar CT reconstructions. There is a lack of information about the 3BL’s real 3D morphology, anatomical variations and their possible classification, posing the question if there is a most common phenotype. A precise assessment of the 3BL and its entry zone into the posterior ethmoid would also lead to a safer dissection. For these reasons, we performed a descriptive CT morphological study of the 3BL’s 3D shape and anatomical features.

## Methods

### Case acquisition

In this retrospective study, non-enhanced cone beam CT scans (CBCT; slice thickness: 0.25 mm) of 25 anonymized patients (50 sides; 16 females and 9 males, mean age: 51.8 years, range: 25.6–83.6 years) performed for dental diagnostics were analyzed with the 3DSlicer software (https://www.slicer.org)^12^. Pediatric cases as well as individuals with sinonasal pathologies (inflammation, polyps, tumor), trauma, surgery in the anamnesis were excluded. Furthermore, CBCTs of 19 formaldehyde-fixed cadaveric heads (38 sides) and 6 skulls (12 sides) provided by the Dept. of Anatomy, Semmelweis University, Budapest, Hungary were investigated. Here, same exclusion criteria were applied. A total of 100 sides have been analyzed.

### Definition of the 3BL

**Figure 1:**
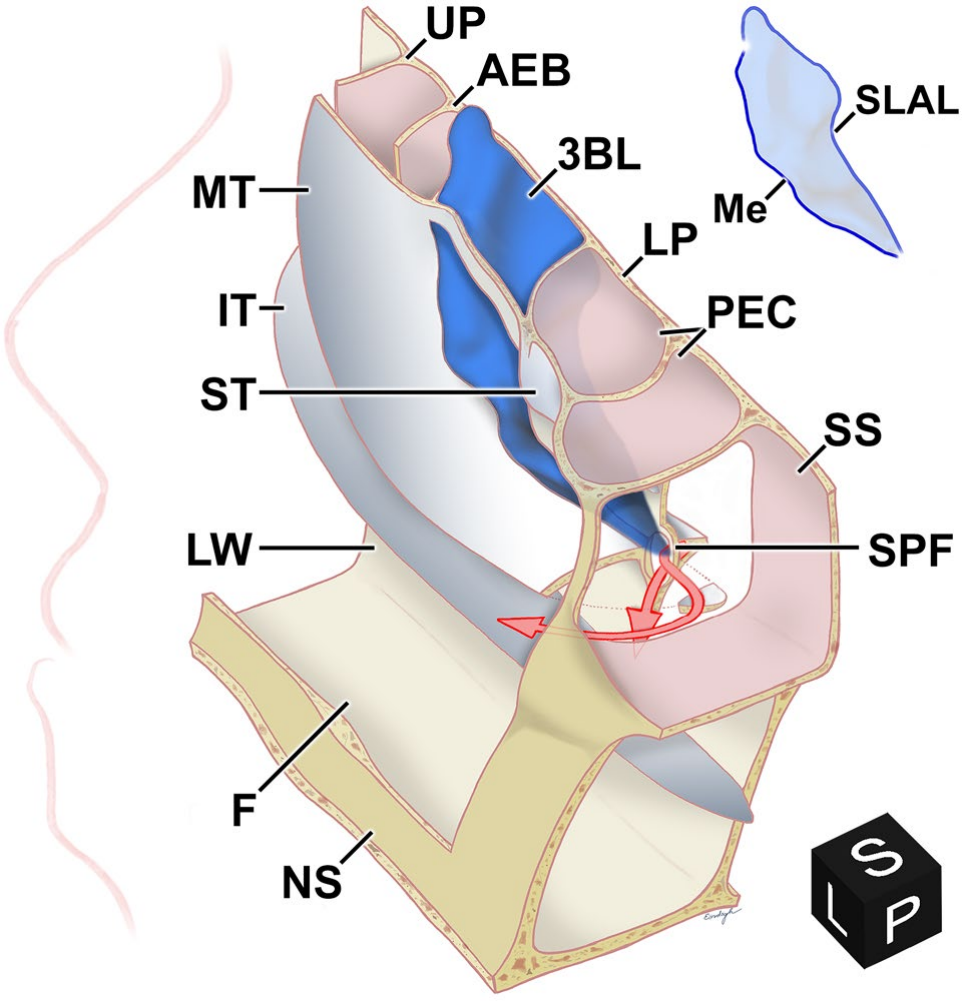
**Topographic anatomy of the basal lamella of the right middle turbinate.** Postero-supero-medial aspect (black cube from the 3DSlicer software depicts the viewing angle—*L* left, *S* superior, *P* posterior) of the right nasal cavity with fenestrated structures. Upper right: silhouette of 3BL. The floor (F) and lateral wall (LW) of the nasal cavity along with the nasal septum (NS), the superior (ST), middle (MT) and inferior turbinate (IT) are depicted. The lamina cribrosa has been removed. The 3BL originates from the superolateral attachment line (SLAL). The medial border (Me) separates the 3BL from the MT. The 3BL separates the anterior (e.g. ethmoidal bulla) and posterior ethmoidal cells (PEC). The uncinate process (UP), the anterior wall of the ethmoidal bulla (AEB), the lamina papyracea (LP), the sphenopalatine foramen (SPF) with the branches of the sphenopalatine artery (red arrows) and the sphenoid sinus (SS) are also illustrated.

The middle turbinate is composed of the *lamina recurvata* (the free turbinate part within the nasal cavity, usually understood as *the* middle turbinate) and the intraethmoidal *lamina basalis* (3BL). We postulated that the 3BL is a circumscribed object with defined borders (Fig. 1). The division relies mainly on the triplanar identification of neighboring structures: the 3BL is surrounded by ethmoidal cells and spaces, contrary to the MT, which is surrounded medially by the nasal cavity.

The *medial border* is the imaginary curve where the 3BL enters the nasal cavity and transitions into the lamina recurvata. The *superior border* of the 3BL is the attachment line on the ethmoidal roof. The *lateral border* is the curvy attachment line on the orbital lamina of the ethmoid bone (lamina papyracea), the medial wall of the maxillary sinus and eventually the medial surface of the palatine bone. The *superior* and *lateral borders* build together the attachment line on the skull base, orbit, maxilla and lateral nasal wall. We call this the *superolateral attachment line* (SLAL). Posteroinferiorly, the medial and lateral borders usually meet in an acute angle; it is mostly found at the region of the ethmoidal crest.

In each case, we created a 3D model of the 3BL using the Segment Editor tool of the 3DSlicer software. Furthermore, we analyzed the triplanar reconstructions. Each case has been revised on a second occasion.

### Parameters of the 3BL

**Figure 2:**
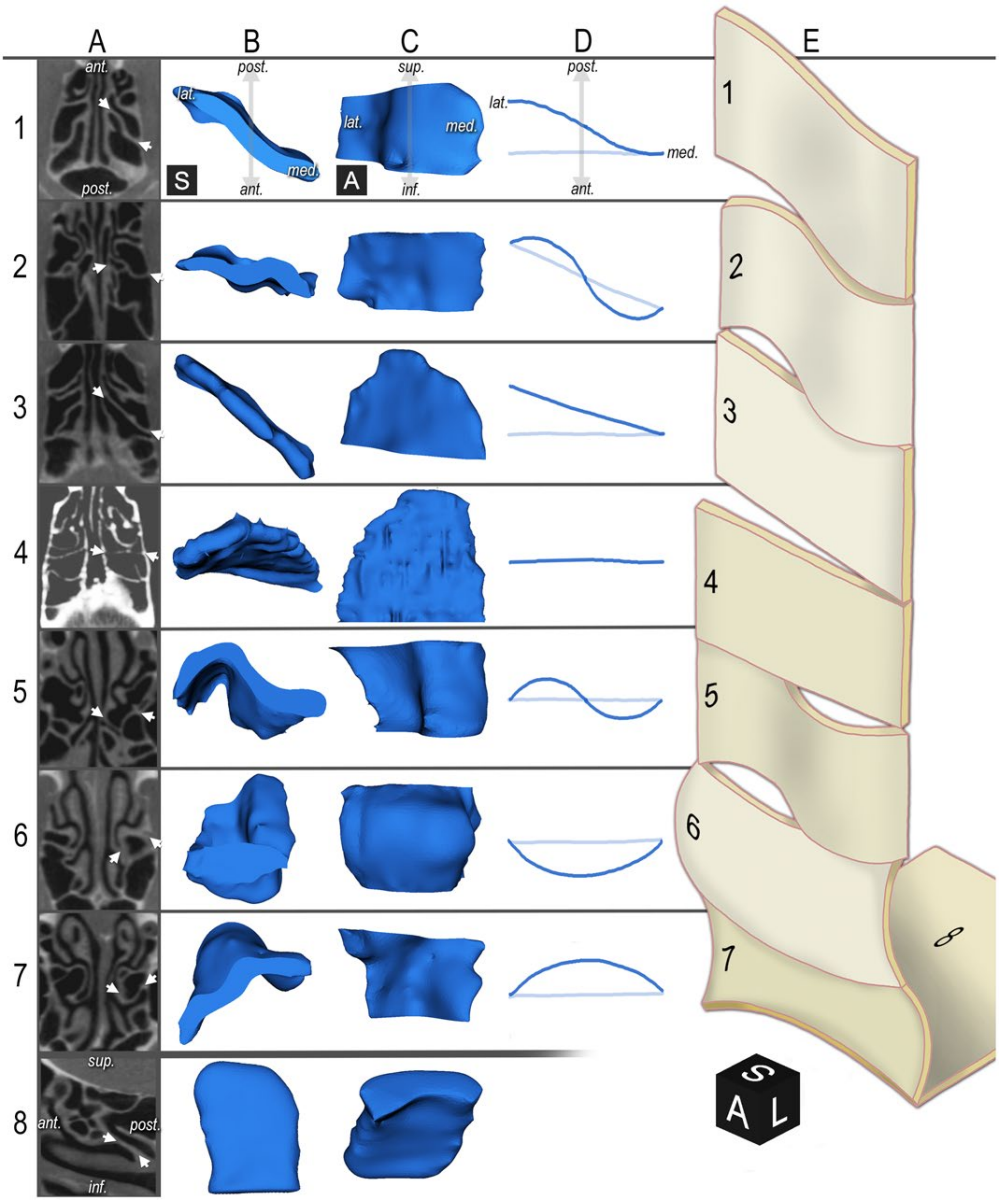
**Examples for the 3rd basal lamella segment types. **Rows distinguish segments types. Columns provide various illustrations. Some images are mirrored for better understanding. Black cubes—*A* anterior, *L* left, *S* superior. 1st row: sigma plate. 2nd row: oblique wave. 3rd row: oblique plate. 4th row: frontal plate. 5th row: frontal wave. 6th row: convex plate. 7th row: concave plate. 8th row: horizontal plate. (For the concave/convex plate, see Fig. 5). Column A: example for CBCT appearance. *Arrows* enclose the 3BL. A1–7: axial view (see A1 for orientation); A8: sagittal view. Column B: superior view of the segments’ 3D shape; see B1 for orientation. Column C: anterior view of the segments’ 3D shape; see C1 for orientation. Column D: axial schematic drawing of the segments’ geometry; see D1 for orientation. Not applicable for the horizontal segment. Column E: 3D schematic drawing of the segment types as observed from supero-antero-medial.

Triplanar CT reconstructions depict the 3BL in 2D from artificially chosen directions. We established a segmenting method to accurately and apparently describe the geometry of each 3BL’s real 3D shape. Segment borders were typically where ethmoidal cells meet. We followed a vertical order from superior to inferior. We distinguished 295 segments of 100 3BLs, categorized into 9 types (Fig. 2).

The *frontal plate* (Fig. 2, 4th row) is basically a 2D plate in the frontal plane. The *frontal wave* (Fig. 2, 5th row) is similar but bears anterior and posterior protrusions and appears on axial images as a waveform. The *oblique plate* (Fig. 2, 3rd row) is basically a 2D plate which angles with the frontal plane. Frontal segments can be easily distinguished from obliques on axial images. The *oblique wave* (Fig. 2, 2nd row) is like the frontal wave but its main axis is oblique. The *sigma plate* (Fig. 2, 1st row) shows a sigmoid (“S”-like) angulation. The *horizontal plate* (Fig. 2, 8th row) is a 2D plate nearly in the horizontal plane. (We apply this nomenclature for didactical reasons.) *Concave* (Fig. 2, 6th row) and *convex plates* (Fig. 2, 7th row) develop due to ethmoidal cell bulging and refer to their shape as observed from anterior. Their combination results in a complex *concave/convex plate*, which will be specified later.

Furthermore, intrinsic parameters of the 3BL models (e.g. surface size) have been defined. We also studied the neighboring anatomical features of the 3BL: the bordering structures; the vertical extension of the part in the ethmoidal fovea (the distance between the horizontal level of the most superior point of the MT where it undergoes into the 3BL *and* the most superior point of the 3BL); the presence of pneumatized middle turbinate as well as the adherence of the ethmoidal bulla and the 3BL.

### Statistical analysis

The statistical analyses have been performed using the R statistical programming language^13^ (version 3.6.0, https://www.R-project.org) with the significance level set at p < 0.05 and confidence interval set at 95%. The continuous variables with normal distribution were compared using paired t-test. When continuous variables were not normally distributed, the Wilcoxon rank test and Mann–Whitney U test were used. The normality of continuous variables was controlled with Shapiro–Wilk test. The categorical variables were evaluated using the Fisher exact test. The association between numerical variables was examined with the Pearson correlation.

### Ethics

All methods and investigations were carried out in accordance with relevant guidelines and regulations. Regulations on the tissue handling of the Dept. of Anatomy, Semmelweis University were accepted by the Senate of the Semmelweis University (“Rector act. 1. R/1/2017 VII.24”). A permission to use the samples was obtained from the institutional responsible of body donation. The CTs were anonymized and performed for other (dental) diagnostics. These belong to the database of the Dept. of Anatomy, Semmelweis University. A permission to use the anonymized images was obtained from the institutional responsible. This study is in accordance with the ethical standards of the institutional and/or national research committee, the regulating law (“1997. year CLIV. law 16. Section 2nd paragraph”) and the 1964 Helsinki declaration and its later amendments or comparable ethical standards. Based on these standards and regulations, for this retrospective study an ethical approval is not necessary. For this type of study, formal consent is not required.

## Results

### Geometry of the 3BL

**Figure 3:**
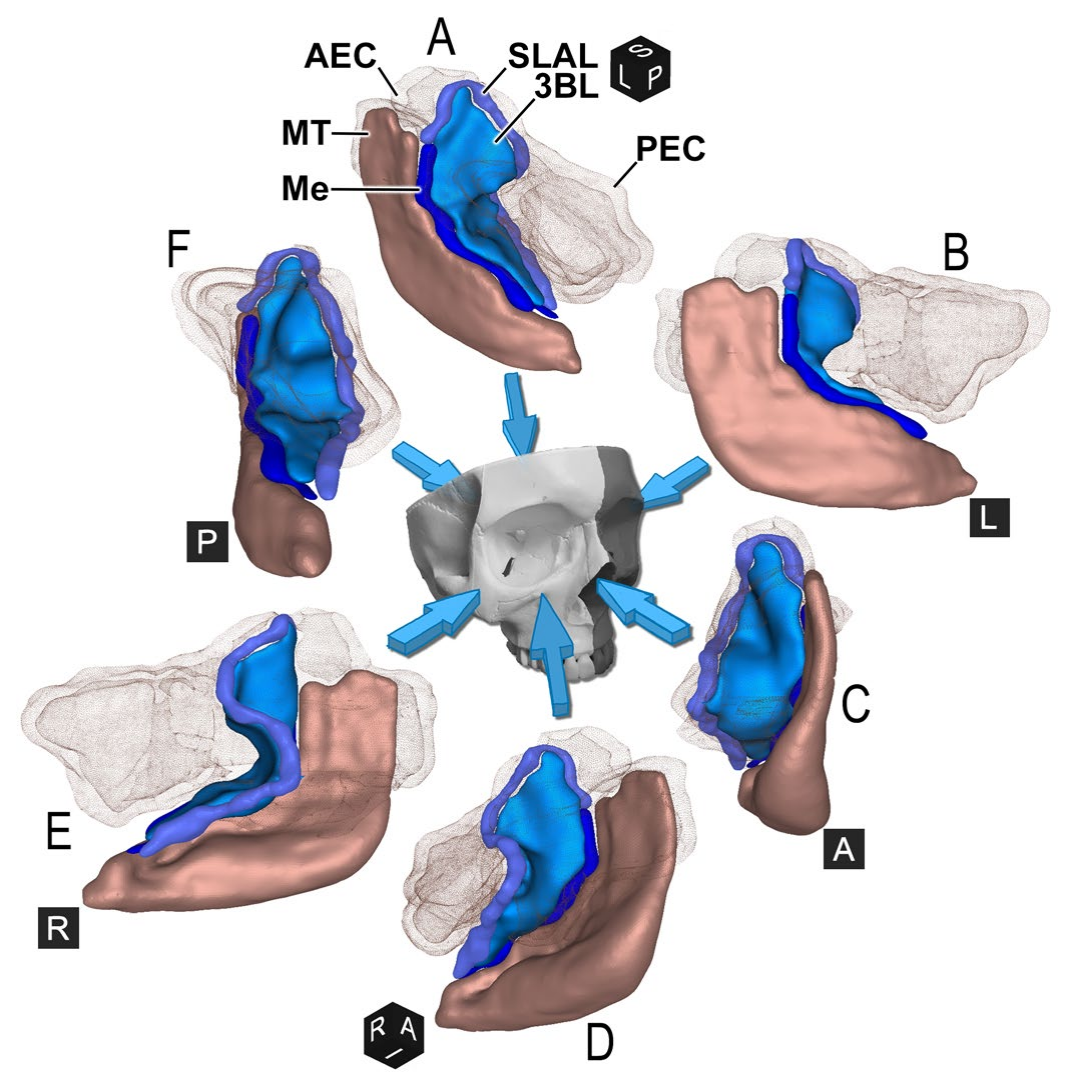
**The basal lamella of the right middle turbinate, observed from various directions. ***Skull with arrows* demonstrate viewing directions. Black cubes—*A* anterior, *I* inferior, *L* left, *P* posterior, *R* right, *S* superior). 3D CBCT reconstruction inspected from supero-postero-medial (**A**), medial (**B**), anterior (**C**), infero-antero-lateral (**D**), lateral (**E**), posterior (**F**). The 3BL originates from the superolateral attachment line (SLAL). The medial border *(Me)* separates the *3BL* from the MT. The punctuated silhouettes demonstrate the anterior (AEC) and posterior ethmoidal cells (PEC).

The 3BL—a thin bone plate with occasional circumscribed thickenings up to 3 mm, covered by a mucous membrane—has been defined in each case (Fig. 3, Video 1). Its average total anterior and/or inferior surface was 394.54 ± 127.78 (range 223.77–835.20) mm^2^ on the left and 399.9 ± 124.69 (range 189.80–824.22) mm^2^ on the right side. Comparing the two sides using paired Wilcoxon test, there was no statistically significant difference (p = 0.3868, CI − 42.15 to 17.61).

**Figure 4:**
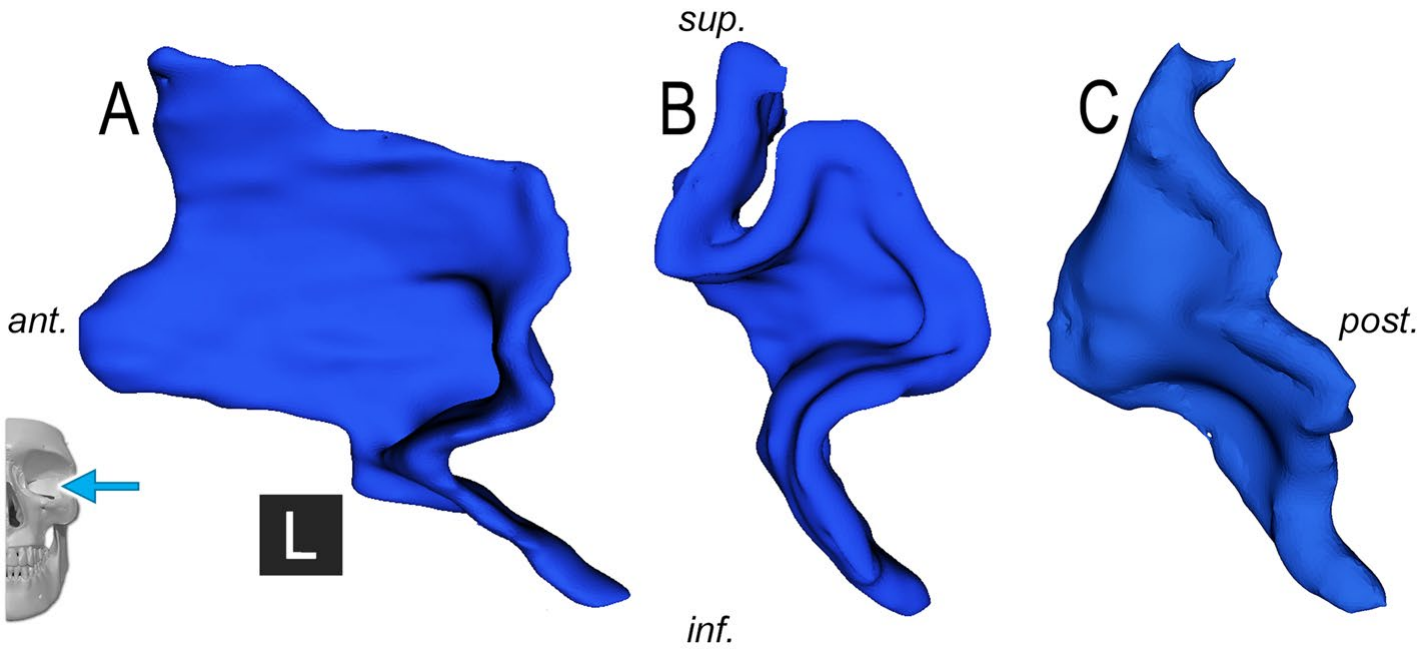
**Examples for the basal lamella of the left middle turbinate, observed from lateral.***Skull with arrow* shows viewing direction. Black cube—*L* left. The conventional basal lamella phenotype of an anterior frontal and a posterior horizontal part is not valid for **A–C**

In general, we could not observe a stringent “L”-shaped 3BL appearance with 2 portions in a lateral view. Our data showed a great diversity of 1–5 different segments (Fig. 4). On the left side the 3BL was built of 2.96 ± 0.67 segments (median: 3; range 2–4); on the right there were 2.94 ± 0.74 (median 3; range 1–5). Overall, there were usually 3 segments without significant side difference (Fisher exact test, p = 0.92). We identified a 2nd segment in 99%, a 3rd in 76%, a 4th in 19% and a 5th in 1% of all sides (Table 2). Therefore, the division of the 3BL to segments is more eligible than to an anterior frontal and a posterior horizontal part. Pearson correlation test showed no association between the number of segments and the surface area neither on the left (R = 0.09, p = 0.51, CI − 0.19 to 0.36) nor on the right side (R = 0.25, p = 0.08, CI − 0.03 to 0.49).

**Table 2:** Localization and prevalence of segment types.

Segment	Localization (sides)					Prevalence (%)	
	1st	2nd	3rd	4th	5th		
N	100	99	76	19	1	100 sides	295 segments
Horizontal		5	44	18	1	68	23.05
Concave/convex	9	49	8	1		67	22.71
Sigma	55	11				66	22.37
Concave	9	17	7			33	11.19
Convex	6	11	13			30	10.17
Frontal plate	8	2				10	3.39
Oblique plate^AM-PL^	7	1				8	2.71
Oblique wave	4	3				7	2.37
Frontal wave			3			3	1.02
Oblique plate^PM-AL^	2		1			3	1.02

Segments counted from superior to inferior. *AM-PL* anteromedial-posterolateral, *PM-AL* posteromedial-anterolateral.

In regard to their prevalence, we separated 3 main segment groups. A cohort of 3 types (horizontal, concave/convex, sigma) was present in 66 or more sides. The horizontal plate was the most frequent (68%). In the residual cases (32%) we observed a vertically oriented (not “L”-shaped) 3BL without a horizontal portion where the posterior MT attached directly to the ethmoidal crest. Examples of the second group (concave, convex) were observed on 30–33 sides. Rare types (10 or less sides) formed the third cohort.

Counting from superior to inferior, the *sigma plate* was the 1st segment in 55 sides, the 2nd in 11 sides but was not found further inferiorly. The *concave/convex* was mostly the 2nd segment (49 sides). The *horizontal plate* was mainly the 3rd (44 sides) or 4th (18 sides), but never the 1st segment, implying to its typical posteroinferior location.

### The most frequent phenotype of the 3BL

The 9 segment types may result in an endless variability of the 3BL. Table 2 already showed the strong presence of a possible 3BL which should be built of the *sigma* (as the 1st), *concave/convex* (2nd) and the *horizontal* segments (3rd).

**Figure 5:**
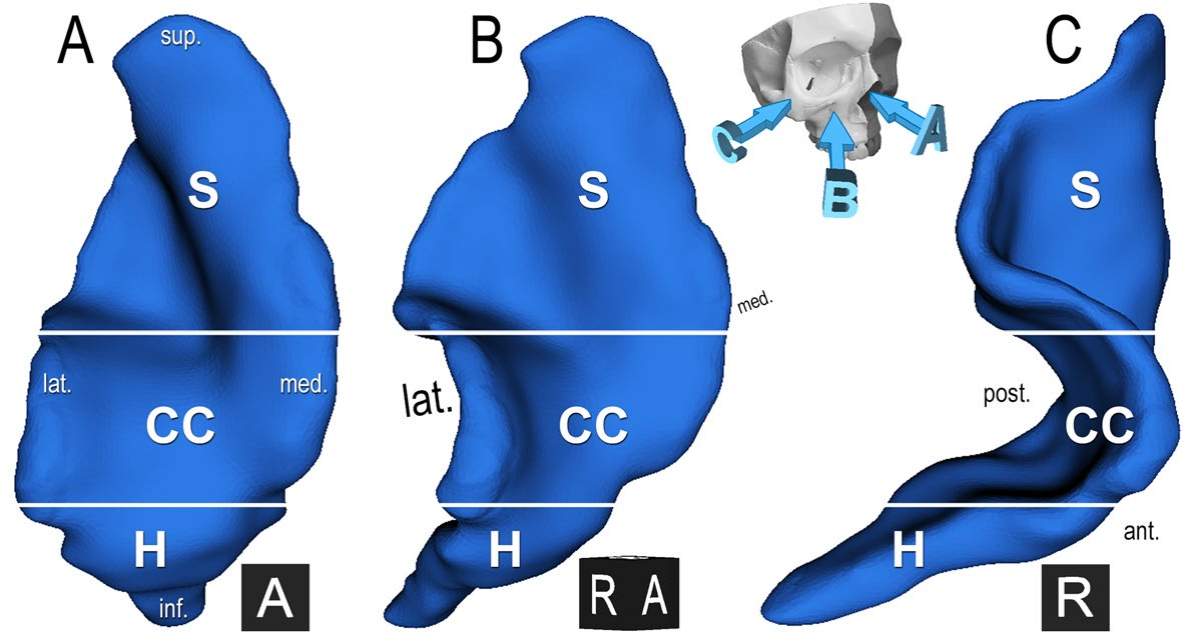
**The most frequent basal lamella phenotype of the middle turbinate, right side.***Skull with arrows* depict viewing directions: anterior (**A**), circa 45° anterolaterally rotated (**B**), lateral (**C**). The sigma (S), concave/convex (CC), horizontal segments (H) can be identified. Black cubes—*A* anterior, *R* right.

Correspondingly, our consecutive analysis showed a high prevalence of two similar phenotypes. We described a *sigma–concave/convex–horizontal* trisegmental 3BL (Table 3, Fig. 5) in 27% and a *sigma–concave/convex* bisegmental 3BL in 9%. Accompanying other segments, the *sigma–concave/convex* bisegmental pattern was described in 4 further cases (4%), the *sigma–concave/convex–horizontal* trisegmental pattern on further 2 sides (2%). Summarily, the *sigma–concave/convex* pattern was present in 42%. 8 individuals (16%) showed bilateral segmentation pattern identity and were all in this cohort. Other patterns had a low prevalence of 1–3%.

**Table 3:** Most frequent patterns of the sigma, concave/convex and horizontal segments.

Segment type				Prevalence (%), N = 100
Sigma	Concave/convex	Horizontal	Other	
✓	✓	✓		27
✓	✓			9
✓	✓		✓	4
✓	✓	✓	✓	2

There are anatomical conditions which are associated with the *sigma* and *concave/convex* segments’ development and explain their high prevalence. The *sigma plate* originates from the posterior expansion of the lateral anterior ethmoidal cells and/or the anterior expansion of the medial posterior ethmoidal cells. It was always anteromedial-posterolateral oriented in our material. The number of participating cells was not constant.

**Figure 6:**
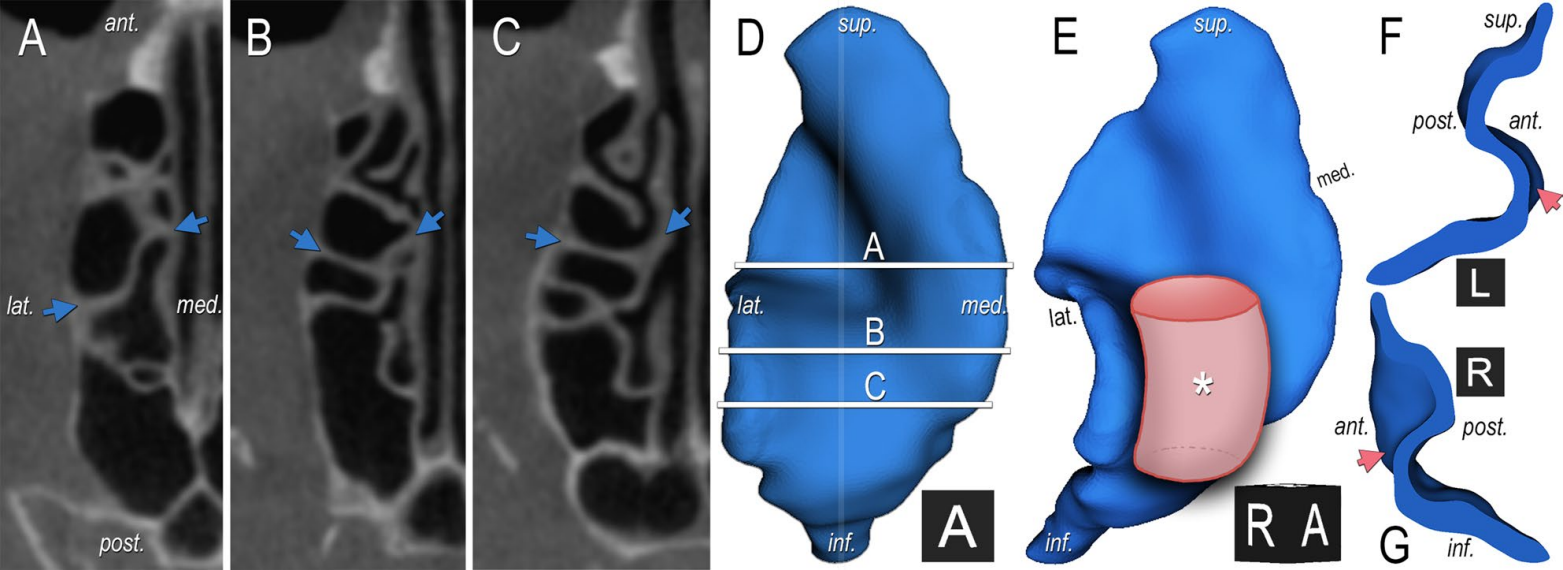
**Generation of the concave/convex segment.** The right ethmoid complex on axial CBCT reconstruction with a sigma (**A**) and a concave/convex segment (**B–C**) of the 3BL enclosed by *blue arrows* (see (**A**) for orientation of **A–C**. **D** Depicts the anterior view of the 3BL’s 3D shape (identical to Fig. 5A); *horizontal lines* match to slices of **A–C**. **E** Depicts the circa 45° anterolaterally rotated view of the 3BL’s 3D shape (identical to Fig. 5B). The 3BL of **D** was split along the *vertical line* to a lateral (**F**) and medial (**G**) part. Black cubes—*A* anterior, *L* left, *R* right. The facilitating factors of the concave/convex segment can be identified (**B,C**): the posterior ethmoid is wider than the anterior; the attachment of the 3BL to the orbital lamina of the ethmoid bone is approximately 90°; the medial 3BL border is slightly anterior compared to the lateral border and the middle part is posterior; a posterior ethmoidal cell produces laterally an anterior shift of the 3BL. Consequently, from an anterior point of view the concave/convex segment seems to be shaped by an object like a “bent tube” (**E**); impact demonstration: *). The cut edge is a convex curvature (**F–G**). On axial images, the shape is concave with anterior protrusions on the sides ((**B–C**,**F–G**) with *pink arrows*).

From an anterior point of view, the *concave/convex* segment appears to be convex on the sagittal images but concave on the axial images, as if it would have been shaped by a “bent tube” object, e.g. the ethmoidal bulla (Fig. 6). Accompanying factors were constantly observed in the presence of this segment type. Firstly, on axial images, compared to the anterior ethmoid, the posterior ethmoid was wider. Secondly, the attachment of the 3BL to the orbital lamina of the ethmoid bone was approximately 90°. Thirdly, on axial analyses, the medial border of the 3BL was located slightly anteriorly compared to its lateral border. The middle part of the 3BL is posteriorly. Lastly, a posterior ethmoidal cell (or infraorbital Haller-cell, as seen on 2 sides) produced laterally an anterior shift of the 3BL. Nota bene: the first two statements exist together.

### Topographic anatomy of the 3BL

The 3BL is bordered laterally by the orbital lamina of the ethmoid bone, the maxillary bone and where appropriate by the perpendicular plate of the palatine bone (Fig. 1, Video 2, “3D model” as Supplementary Material). In 3%, the maxillary sinus is separated from the 3BL by an infraorbital (Haller-) cell. Discontinuities are very uncommon.

The vertical extension of the superior part in the region of the ethmoidal fovea was 4.90 ± 2.06 (range 0–10.90) mm on the left and 5.10 ± 1.75 (range 0–9.25) mm on the right side. There was no significant difference between both sides using paired t-test (p = 0.47, CI − 0.74 to 0.35).

Pneumatized MT (concha bullosa) led to deformations and increased surface of the 3BL. The average surface area of the 3BLs was 380.14 ± 111.99 mm^2^ (189.80–824.22) in cases without and 443.41 ± 149.44 mm^2^ (218.20–835.21) in cases with concha bullosa. The difference between these groups was statistically significant using Mann–Whitney U test (p = 0.04, CI 1.17–119). Pneumatization originating from the anterior ethmoid was seen in these cases bilaterally in 14%, unilaterally on the left side in 4%, on the right in 5%. Pneumatization from the posterior ethmoid was seldom (2%, 3%, 0%, respectively). Comparing the prevalence of pneumatized MT on the two sides using Fisher exact test, the difference was statistically not significant (p = 0.45).

The 3BL partly comprised the posterior wall of the ethmoidal bulla in 38 cases bilaterally, on 6 individuals on the left and 5 cases on the right side (87% of all sides). On 4 sides among them, a posterior ethmoidal cell with anterior bulging mimicked the ethmoidal bulla which was hypoplastic in these cases. On 13 sides (12 individuals), there were no attachment to the ethmoidal bulla.

## Discussion

Stammberger et al.^7,14^ described the 3BL as an “L”-shaped bone plate in a lateral view with an anterior frontal and a posterior horizontal part, connecting the MT to the skull base and thus stabilizing it. As a result of the ethmoidal cells’ expansion and distortion, each 3BL is unique^10^. Consequently, the intraoperative identification of this major landmark structure can be challenging despite thorough studies of CTs prior surgery. Recently the senior author provided a comprehensive overview of the ethmoidal anatomy^15^. However, there are only a few morphological analyses about the 3BL.

According to Kim et al.^8^, the 3BL might have the most uniform shape among the basal lamellas. They distinguished “the anterior portion […] in an almost frontal” (vertical) and “the posterior portion […] in an almost horizontal plane”, similarly to the already mentioned “L”-shape. In their cadaveric-radiological study, the anterior portion attached to the skull base on a straight (91%) or anteriorly indented (9%) line. They categorized the relationship between the 3BL and the ethmoidal bulla whether the posterior bulla wall is the lower half of the anterior vertical portion of the 3BL (38%), or the posterior bulla wall builds the entire anterior portion of the 3BL (21%). Similar to them, we frequently (87%) found an adhesion.

Based on the bilateral analysis of 100 CTs, Wu et al.^16^ observed an oblique posterior plane with circa 127° angle to the anterior part. They described the horizontal portion as a “slope”. On our sophisticated 3D models, one could only broadly assess this angulation. They separated linear (22%), curved (56%) and angular (20%) appearances of the 3BL as observed on axial images. These are of great clinical value but remain artificially chosen data extracts which only partly imply to the real shape.

A shortcoming of the studies mentioned above is the subjective, schematizing and simplified description of the 3BL’s intricate and diverse morphology. In this analysis we aimed to demonstrate the 3BL’s 3D anatomy. We analyzed 100 basal lamellas and identified phenotypes built up from 1 to 5 pieces of 9 distinctive segment types. Our results confirm the general diversity of the 3BL. However, the high occurrence of the *sigma–concave/convex–horizontal* 3BL (27%) and the *sigma–concave/convex* pattern (42%) may imply to a typical phenotype which we believe to be a novel information. Contrary to most reports, we could not recognize the “L”-shape of the 3BL in a lateral view. In accordance with many authors, we identified a basically vertical anterior part of the 3BL. However, after that we observed numerous segmentation patterns here. The horizontal portion lacked in 32%. Consequently, the present study showed that the commonly accepted schematic shape of the 3BL is not generally valid. This made a new classification necessary.

Diverse methods and software solutions were considered; only CBCTs (or high resolution CTs) with the 3DSlicer application enabled precise, reproducible and standardized evaluation. We sought for resemblance to geometrical forms which is certainly no absolute identity. It has to be noted that we focused on the appearance of the 3BL from an anterior point of view as this is the common direction of any surgical approach. Nevertheless, revisions and analysis of accompanying anatomical factors support our findings.

We are not aware of any other 3DSlicer-based surgically oriented studies of the middle turbinate’s anatomy. However, diverse workgroups already reported on this software’s feasibility to analyze the paranasal sinuses and the skull base for anatomical^17,18^, biophysical^19,20^ or forensic^21^ purposes. Raappana et al. described a 3DSlicer-based planning workflow for transnasal pituitary surgery, focusing on the relevant parasellar anatomy^22^.

Dissection within the ethmoidal labyrinth should be performed with an inferomedially accented orientation to avoid unintended exposure of the anterior cranial fossa superiorly and the orbit laterally. For this reason, the segments can be reliable surgical landmark structures, especially the *sigma*, the *concave/convex* and *horizontal* types which showed a high prevalence in our data. The description of the variations with a focus on the *sigma* and *concave/convex* segments may be of particular clinical significance as they belong to the anterior vertical part of the 3BL which is the typical surgical dissection site. Both examples have a well-recognizable phenotype. The *sigma plate* was always anteromedial-posterolateral oriented and appeared to be the 1st or 2nd segment from superior (55% and 11%, respectively). Consequently, dissection of the *sigma plate* likely leads to the superior part of the posterior ethmoid. The *concave/convex* was the 2nd segment in 49% but the 1st in 9%. According to our data, dissection of the *concave/convex* segment likely leads to the inferior part of the posterior ethmoid. Thus, the *concave/convex* segment’s peculiar shape may be an additional information besides other well-known anatomical landmarks to define the pathway to the posterior ethmoid.

The triplanar analysis of axial, sagittal and frontal 2D reconstructions is an efficient method and routinely applied in the daily care. Commonly, the 3BL is understood as a continuous structure with a variety of curvatures without definitive angles, which could be a criticism to the statements of the present work. Our study is based on this widely accepted concept, but additionally represents how exactly the 3BL’s “fluid” shape can be derived from the accompanying ethmoidal cells and which factors influence the 3BL’s shape. We hope that this descriptive manuscript provides a better understanding of this delicate anatomical area for morphologists and surgeons. We believe that the 3D depiction of the basal lamella enables not only to understand its true shape but to accurately interpret the triplanar 2D reconstructions and assess the lamella’s and the neighboring structures’ topography. CT analysis prior to surgery can help to identify the desired entry zone and separate it from the superior and horizontal portions of the 3BL. The segmenting method is eligible, as it relies on the simple observation that the borders of segments are also the borders of ethmoidal cells. We identified a 3BL-phenotype with notably high prevalence and strong relation to given accompanying factors, serving as the major observation of this study. We suggest to choose a surgical approach after the geometrical details of the ethmoid were configured beforehand. However, the clinical impact of our findings has to be confirmed by surgeons and needs further research.

## Conclusion

The 3BL is an important landmark for endoscopic sinus and skull base surgery with variable morphology. Studies using 3D reconstructions gained from CT scans allow a precise description of the 3BL. We introduced a segmental anatomical description which is more applicable than dividing them into an anterior frontal and a posterior horizontal part. The *sigma–concave/convex–horizontal* trisegmental and the *sigma–concave/convex* bisegmental pattern were common suggesting their close relation to the typical 3BL phenotype. This study can help surgeons to predict anatomical variations during endoscopic sinus and skull base procedures and improve their intraoperative orientation during ethmoidal dissection.

## Supplementary Information

Supplementary Information 1.

Supplementary Information 2.

Supplementary Video 1.

Supplementary Video 2.

## Abbreviations

2D: Two-dimensional
3D: Three-dimensional
3BL: Basal lamella of the middle turbinate (3rd basal *or* ground lamella)
CBCT: Cone beam computed tomography
CT: Computed tomography
MT: Middle turbinate of the nasal cavity
SLAL: Superolateral attachment line
