# Supplementary material for: Three-dimensional structure of the basal lamella of the middle turbinate
Source: Sci Rep. 2021 Sep 9;11:17960. doi: 10.1038/s41598-021-97331-2 (PMC8429674; doi:10.1038/s41598-021-97331-2)
Supplement: Supplementary file 2 — Supplementary Information 2. [file 41598_2021_97331_MOESM2_ESM.docx]

**Video 1.**

Video 1 shows an example for the basal lamella of the middle turbinate.

**Video 2., 3D model**

Video 2 demonstrates the topographic anatomy of the basal lamella of the middle turbinate (case of Video 1). For demonstrative purposes, some structures have been virtually dissected. **Yellow:** bone. **Blue:** basal lamella of the middle turbinate. **Light brown:** middle turbinate. **Dark brown:** mucosa of the nasal cavity, maxillary sinus and nasopharynx. **Light green:** anterior ethmoidal cells. **Darker green:** posterior ethmoidal cells. **Darkest green:** sphenoid sinus. **Orange:** pituitary tumor.

Please find also the corresponding 3D model (supplementary material: “Model.pdf”). Elements can be freely removed.
